# Olivia Newton-John Cancer Research Institute 10-year Anniversary Conference – a decade of discovery and translation

**DOI:** 10.1038/s41419-025-07612-1

**Published:** 2025-04-09

**Authors:** Katherine Eljammas, Tianming A. Li, Liam Neil, Rebecca Nightingale, Farrah El-Saafin, Jean Berthelet, Lisa A. Mielke, Bhupinder Pal

**Affiliations:** 1 Olivia Newton-John Cancer Research Institute, Heidelberg, VIC Australia; 2 School of Cancer Medicine, La Trobe University, Heidelberg, VIC Australia; 3 La Trobe Institute for Molecular Science, La Trobe University, Bundoora, VIC Australia

**Keywords:** Cancer, Medical research

The Olivia Newton-John Cancer Research Institute (ONJCRI) celebrated its 10th anniversary in September 2024 with a two-day event featuring an academic conference. The first day began with opening remarks by Professor Marco Herold (CEO) and Dr. Christine De Nardo (COO), emphasising ONJCRI’s commitment to advancing effective, tolerable, and equitable cancer care (Fig. 1A). Hon. John Brumby AO (Officer of the Order of Australia), Chancellor of La Trobe University and Founding Chair of the ONJCRI Board, joined the Hon. Mary-Anne Thomas MP, Health Minister of Victoria (Australia), and Professor John Mariadason (ONJCRI) to reflect on ONJCRI’s founding and its strategic partnership with La Trobe University, where the institute serves as the School of Cancer Medicine. Dr. Lisa Briggs, a lung cancer survivor, highlighted the critical role of consumers in ONJCRI’s journey, sharing her story and the successes of the TRACKER biobank lung cancer consortium (MRFCRI000092).

**Fig. 1:**
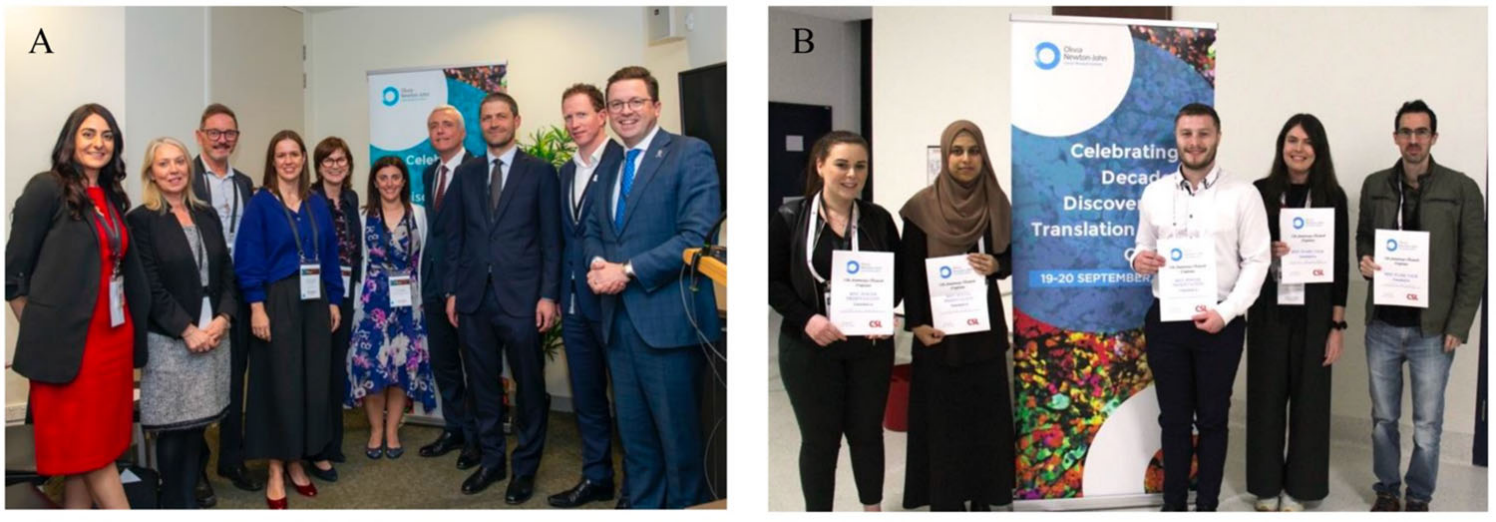
ONJCRI 10-year Anniversary Conference. **A** Invited guests at the welcome event. **B** Winners of best oral and poster presentations.

The second day featured scientific presentations showcasing groundbreaking research in cancer biology, therapies, and clinical trials.

## Aquaporin 5 (AQP5): Unveiling new pathways in gastric carcinogenesis

Keynote speaker, Professor Nick Barker (A*STAR Institute of Molecular and Cell Biology, Singapore) presented his research on the role of Leucine-rich repeat-containing G-protein coupled receptor 5 **(**LGR5) in gastric stem cells, a critical factor required for epithelial self-renewal and is linked to gastric carcinogenesis. LGR5^+^ stem cells uniquely co-expresses AQP5 within the pyloric gland bases, where gastric cancers originate. AQP5-expressing cells in nascent gastric cancers were shown to function as a cancer stem-like cell population using lineage tracing, transplantation and targeted ablation assays. AQP5 itself was shown to confer key cancer traits on gastric tumour cells, promoting cancer growth and invasion via modulation of cell proliferation and migration. In human gastric cancers, AQP5 is expressed in primary intestinal and diffuse gastric cancer subtypes, including metastases. Thus, highlighting AQP5 as a potential marker for early cancer detection.

## Exploring mammary epithelial subsets for targeted breast cancer therapies

Professor Jane Visvader (Walter and Eliza Hall Institute (WEHI), Australia) highlighted the role of luminal progenitor (LP) cells in mammary gland development and breast carcinogenesis linked to BRCA2 mutations. Her team has demonstrated that hormone receptor-positive LP cells are highly dynamic in terminal end buds, driving ductal elongation during mammary morphogenesis. In *BRCA2*-mutant breast cancers, a subset of LP cells was found to have aberrant growth properties and dysregulated gene expression, including hyperactivation of mTORC1 signalling. Targeting this pathway in a preclinical model significantly delayed tumorigenesis, with implications for a potential prevention strategy for *BRCA2* mutation carriers.

Dr. Michael Milevskiy (WEHI) presented a 3D epigenetic atlas of mammary epithelial cells, highlighting opportunities for more precise targeting of LP cells in breast cancer where they are emerging as important cells of origin of cancer.

## Decoding the role of BRCA1 mutations in PARPi therapy resistance

Dr Ksenija Nesic (WEHI) presented the mechanisms underlying PARP inhibitor resistance in homologous recombination DNA repair (HRR)-deficient ovarian cancers. Her study identified secondary splice site mutations that drive BRCA1 exon skipping, producing truncated proteins that contribute to PARP inhibitor resistance. These mutations, detected in resistant tumours, underscore the importance of clinical monitoring to optimise treatments to overcome resistance.

## Pioneering new frontiers in glioblastoma and brain cancer treatment

Professor Andrew Scott (ONJCRI) highlighted advancements in targeted cancer therapies and tumour imaging, including discoveries from his laboratory, which have led to the formation of the Centre for Research Excellence in Brain Cancer and the ACRF Centre for Precision Medicine. His team has identified new treatment targets and imaging probes, progressing to phase I/II/III trials in glioblastoma patients. Antibody KB004 (Ifabotuzumab), targeting EphA3, has shown promising results in Phase I trials in leukaemia and glioblastoma patients. Another antibody-drug conjugate (ABT-414, Depatuxizumab Mafodotin) targeting the epidermal growth factor receptor (EGFR), also demonstrated durable therapeutic effects.

## Exploring breast cancer heterogeneity in metastasis

Professor Delphine Merino (ONJCRI) shared insights into breast cancer metastasis mechanisms using optical barcoding to trace clonal origins across diverse tumour microenvironments. Her research revealed that certain cancer cells are intrinsically programmed for metastasis and identified unique adaptations in these cells, presenting new opportunities for targeted therapeutic strategies.

## Advancing cancer care through innovative technologies

Professor Brian Abbey (La Trobe University, Australia) presented a novel technology, his company AlleSense, is currently commercialising as a class II in-vitro diagnostic (IVD) for early cancer detection. By modifying the surface of conventional microscope slides at the nanoscale, this technology leverages surface plasmon resonance (SPR) to enhance light interactions with biological tissues. This enhancement causes cancerous cells to exhibit distinct colour changes compared to normal cells, enabling pathologists to identify them without the need for additional staining. Developed in collaboration with the ONJCRI, the NanoMslide technology has demonstrated strong concordance with traditional immunohistochemistry, thus opening new avenues for advancements in digital pathology.

Dr. Jessica Da Gama Duarte (ONJCRI) presented an Opal multiplex immunohistochemistry panel for analyzing tertiary lymphoid structures (TLSs) in tumours, highlighting their role in anti-tumour immunity. This protocol uses specific markers to grade TLS maturation, offering insights into immune cell coordination and movement within tumours as cancer advances.

Professor Nilmini Wickramasinghe (Optus Chair of Digital Health, La Trobe University) discussed the potential of artificial intelligence to advance personalised cancer care, through “Digital Twins”— a mathematical model that replicates a patient’s symptoms, medical history, multi-omics, along with cohort data to simulate and optimise treatment outcomes. Trials in the U.S. and Australia, including ONJCRI, are currently exploring this technology to improve clinical decision-making and patient outcomes in oncology.

## TP53 plays a critical role in gastric cancer metastasis

Dr. Moritz Eissmann (ONJCRI) showed that mutations in Kirsten rat sarcoma virus (KRAS) and phosphoinositide 3-kinase (PI3K) initiate gastric tumour formation, while tumour protein 53 (TP53) mutations drive progression to distal metastasis. His team discovered that TP53 mutations cause a cytokine dependency shift from interleukin 11 (IL-11) to interleukin 6 (IL-6), promoting aggressive spread. This presents potential for therapies targeting TP53-mutant gastric cancers to limit metastasis.

## Gut microbiomes as a predictor for immunotherapy success

Dr. Ashray Gunjur (Wellcome Sanger Institute, UK) concluded the symposium by discussing key findings from the ONJCRI-led Rare Cancers trial (NCT02923934), which provided immune checkpoint blockade (ICB) therapy to rare cancer patients. Remarkably, about 25% of participants had significant responses to this treatment. Additionally, his team identified gut microbiome signatures linked to positive ICB outcomes across various cancers, indicating the microbiome’s potential as a predictor of treatment success. These findings, reinforced by international studies, underscore a promising shift in research toward optimising ICB treatments based on patient microbiome profiles rather than cancer type alone.

## Cancer Australia’s Proposal for National Framework for Genomics in Cancer Control

Carolyn Der Vartanian (Cancer Australia) outlined the National Framework for Genomics in Cancer Control, a core initiative of the Australian Cancer Plan. Developed through stakeholder consultation, the Framework seeks to integrate genomics into cancer care equitably and safely, ensuring consistent, culturally appropriate, evidence-based applications to address disparities and improve outcomes across the care continuum.

In conclusion, the conference brought together clinicians, scientists, consumers, and parliamentarians to celebrate a decade of ONJCRI achievements, while showcasing recent advancements in cancer research and care. Outstanding presentations by post-graduate and post-doctoral researchers were recognised (Fig. 1B), underscoring ONJCRI’s commitment to fostering future cancer research talent.

